# 2-Amino-3-carb­oxy­pyridinium perchlorate

**DOI:** 10.1107/S1600536812018922

**Published:** 2012-05-02

**Authors:** Fadila Berrah, Sofiane Bouacida, Hayet Anana, Thierry Roisnel

**Affiliations:** a Laboratoire de Chimie Appliquée et Technologie des Matériaux (LCATM), Université d’Oum El Bouaghi 04000, Algeria; b Unité de Recherche de Chimie de l’Environnement et Moléculaire Structurale (CHEMS), Faculté des Sciences Exactes, Université Mentouri Constantine 25000, Algeria; c Centre de Difractométrie X, UMR 6226 CNRS Unité Sciences Chimiques de Rennes, Université de Rennes I, 263 Avenue du Général Leclerc, 35042 Rennes, France

## Abstract

The asymmetric unit includes two crystallographically independent equivalents of the title salt, C_6_H_7_N_2_O_2_^+^·ClO_4_^−^. The cations and anions form separate layers alternating along the *c* axis, which are linked by N—H⋯O, O—H⋯O and C—H⋯O hydrogen bonds into a two-dimensional network parallel to (100). Further C—H⋯O contacts connect these layers, forming a three-dimensional network, in which *R*_4_^4^(20) rings and *C*_2_^2^(11) infinite chains can be identified.

## Related literature

For structural studies of hybrid compounds of 2-amino­nicotinic acid, see: Akriche & Rzaigui (2007 ▶); Berrah *et al.* (2011*a* ▶,*b* ▶). For related perchlorate compounds, see: Toumi Akriche *et al.* (2010 ▶); Bendjeddou *et al.* (2003 ▶). For hydrogen-bond motifs, see: Etter *et al.* (1990 ▶); Grell *et al.* (1999 ▶).
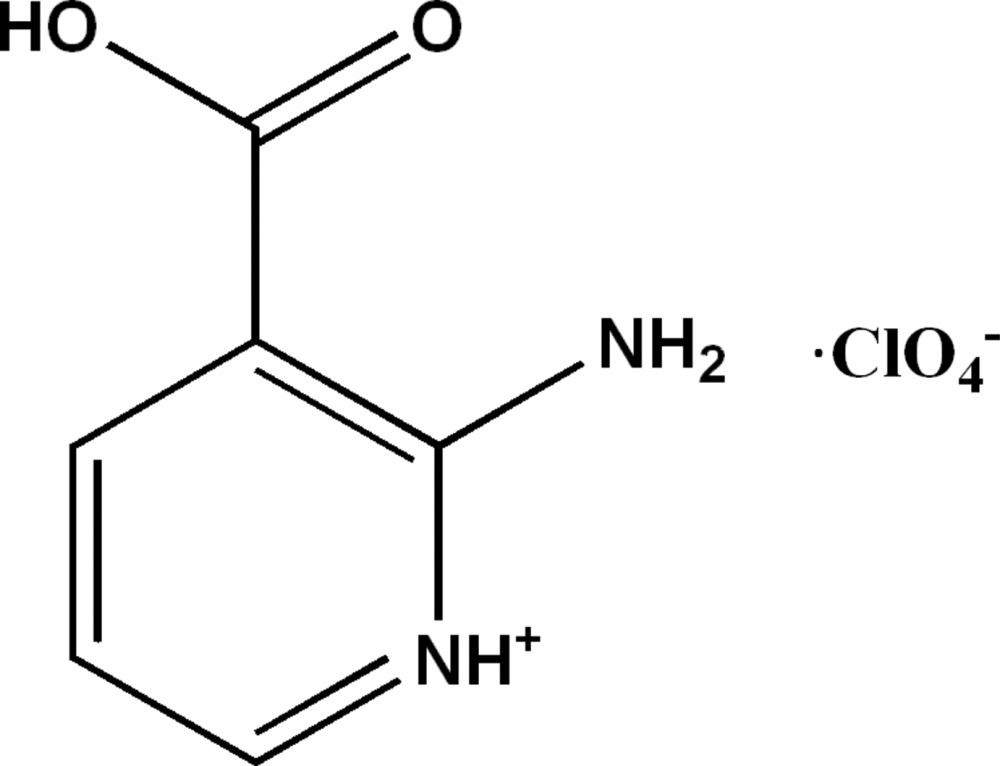


## Experimental

### 

#### Crystal data

C_6_H_7_N_2_O_2_^+^·ClO_4_^−^
*M* *_r_* = 238.59
Monoclinic, 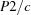

*a* = 17.3573 (12) Å
*b* = 5.0800 (4) Å
*c* = 21.6293 (17) Å
β = 107.239 (2)°
*V* = 1821.5 (2) Å^3^
*Z* = 8
Mo *K*α radiation
μ = 0.43 mm^−1^
*T* = 150 K
0.48 × 0.17 × 0.08 mm

#### Data collection

Bruker APEXII diffractometer
Absorption correction: multi-scan (*SADABS*; Sheldrick, 2002 ▶) *T*_min_ = 0.847, *T*_max_ = 0.966
13822 measured reflections
4142 independent reflections
3305 reflections with *I* > 2σ(*I*)
*R*_int_ = 0.044

#### Refinement

*R*[*F* ^2^ > 2σ(*F* ^2^)] = 0.049
*wR*(*F* ^2^) = 0.110
*S* = 1.11
4142 reflections
273 parameters
H-atom parameters constrained
Δρ_max_ = 0.34 e Å^−3^
Δρ_min_ = −0.40 e Å^−3^

### 

Data collection: *APEX2* (Bruker, 2006 ▶); cell refinement: *SAINT* (Bruker, 2006 ▶); data reduction: *SAINT*; program(s) used to solve structure: *SIR2002* (Burla *et al.*, 2005 ▶); program(s) used to refine structure: *SHELXL97* (Sheldrick, 2008 ▶); molecular graphics: *ORTEP-3 for Windows* (Farrugia, 1997 ▶) and *Mercury* (Macrae *et al.*, 2006 ▶); software used to prepare material for publication: *WinGX* (Farrugia, 1999 ▶).

## Supplementary Material

Crystal structure: contains datablock(s) global, I. DOI: 10.1107/S1600536812018922/ld2056sup1.cif

Structure factors: contains datablock(s) I. DOI: 10.1107/S1600536812018922/ld2056Isup2.hkl

Supplementary material file. DOI: 10.1107/S1600536812018922/ld2056Isup3.cml

Additional supplementary materials:  crystallographic information; 3D view; checkCIF report

## Figures and Tables

**Table 1:** Hydrogen-bond geometry (Å, °)

*D*—H⋯*A*	*D*—H	H⋯*A*	*D*⋯*A*	*D*—H⋯*A*
O1*A*—H1*A*⋯O21	0.82	1.99	2.810 (3)	173
O1*B*—H1*B*⋯O42	0.82	1.96	2.779 (3)	176
N2*A*—H2*A*⋯O32	0.86	2.24	2.968 (3)	142
N2*B*—H2*B*⋯O31	0.86	2.31	3.005 (3)	138
N2*B*—H2*B*⋯O22^i^	0.86	2.34	2.992 (3)	133
N1*A*—H11*A*⋯O22^ii^	0.86	2.50	3.211 (3)	141
N1*A*—H11*A*⋯O32	0.86	2.58	3.231 (3)	133
N1*B*—H11*B*⋯O31	0.86	2.32	3.000 (3)	136
N1*B*—H11*B*⋯O41^iii^	0.86	2.54	3.268 (3)	143
N1*A*—H12*A*⋯O2*B*^ii^	0.86	2.19	2.928 (3)	144
N1*B*—H12*B*⋯O2*A*^iii^	0.86	2.22	2.971 (3)	145
C4*A*—H4*A*⋯O11^iv^	0.93	2.57	3.312 (3)	137
C4*B*—H4*B*⋯O32^v^	0.93	2.53	3.433 (3)	165
C5*A*—H5*A*⋯O11^vi^	0.93	2.37	3.277 (3)	164
C5*B*—H5*B*⋯O12^vii^	0.93	2.52	3.450 (3)	177

Symmetry codes: (i) 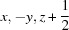; (ii) 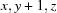; (iii) 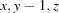; (iv) 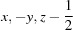; (v) 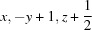; (vi) 
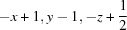
; (vii) 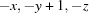.

**Fig. 1.**
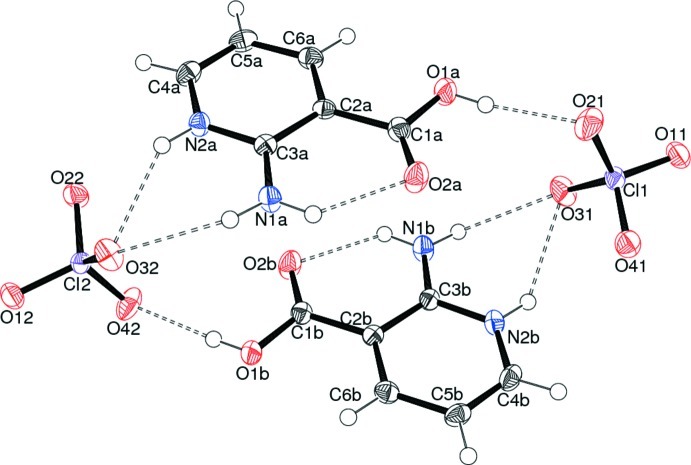
asymmetric unit of the title compound with the atomic labelling scheme. Displacement are drawn at the 50% probability level. Hydrogen bonds are shown as dashed lines.

**Fig. 2.**
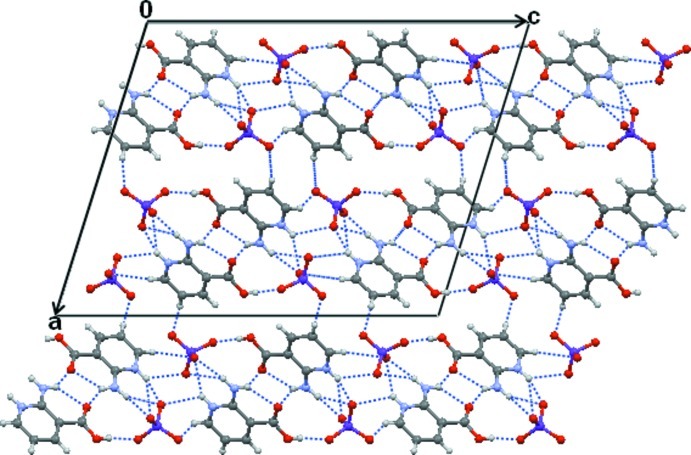
A view parallel to (010) showing cationic and anionic layers alternation along the c axis. Hydrogen bonds are shown as dashed lines.

**Fig. 3.**
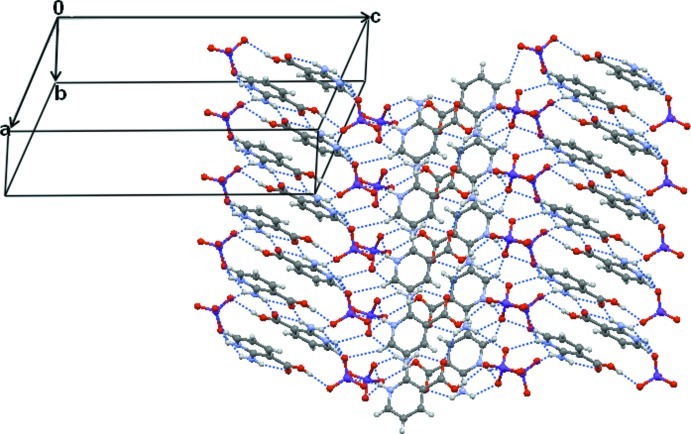
A view of the two-dimensional network showing how dimers are stacked within cationic layers. Hydrogen bonds are shown as dashed lines.

## supplementary crystallographic information

### Comment

As a continuation of the systematic studies on synthesis and structural characterization of the products of derivatives of nicotinic acid with inorganic acids, and as an attempt to establish a relationship between the nature of the anion and the resulting hydrogen-bonding pattern, we report here the crystal structure of the title compound obtained by reaction between 2-aminonicotinic and perchloric acids. Related compounds obtained with dihydrogen phosphate, sulfate and nitrate anions, have been reported previously (Akriche & Rzaigui 2007; Berrah *et al.* 2011*a*,b).

The dimers of 2-aminonicotinium cations are formed *via* N—H···O h-bonds (NH of the amine group with the O of the carboxylic group). Similar dimers have been also observed in the structures with dihydrogen phosphate and sulfate anions (Akriche & Rzaigui 2007; Berrah *et al.* 2011*a*), while cations in the nitrate structure adopt a different configuration (Berrah *et al.* 2011*b*).

In the crystal structure, cationic and anionic layers alternate along the *c* axis and are linked by intermolecular N—H···O, O—H···O and weak C—H···O hydrogen bonds (see table 1) resulting in a two-dimensional network parallel to (100) (Fig.2). Further C—H···O contacts connect these layers, forming a three-dimensional network in which *R*_4_^4^(20) rings and C_2_^2^(11) infinite chains are generated (Etter *et al.* 1990; Grell *et al.* 1999).

### Experimental

Colourless crystals of compound (I) were grown by slow evaporation of an aquoes solution of 2-amino-pyridine-3-carboxylic acid and perchloric acid in an 1:1 stoichiometric ratio.

### Refinement

All H atoms were located in a difference Fourier maps but introduced at calculated positions and treated as riding on their parent atoms (C,*N* or O) with C—H = 0.93 Å,*N*—H = 0.88 Å and O—H = 0.82 Å with *U*_iso_(H) = 1.2 *U*_eq_(C or N) and *U*_iso_(H) = 1.5 *U*_eq_(O).

### Crystal data

**Table:**

C_6_H_7_N_2_O_2_^+^·ClO_4_^−^	*F*(000) = 976
*M**_r_* = 238.59	*D*_x_ = 1.74 Mg m^−^^3^
Monoclinic, *P*2/*c*	Mo *K*α radiation, λ = 0.71073 Å
Hall symbol: -P 2yc	Cell parameters from 3823 reflections
*a* = 17.3573 (12) Å	θ = 2.5–27.4°
*b* = 5.0800 (4) Å	µ = 0.43 mm^−^^1^
*c* = 21.6293 (17) Å	*T* = 150 K
β = 107.239 (2)°	Stick, colourless
*V* = 1821.5 (2) Å^3^	0.48 × 0.17 × 0.08 mm
*Z* = 8	

### Data collection

**Table:**

Bruker APEXII diffractometer	3305 reflections with *I* > 2σ(*I*)
Graphite monochromator	*R*_int_ = 0.044
CCD rotation images, thin slices scans	θ_max_ = 27.5°, θ_min_ = 2.0°
Absorption correction: multi-scan (*SADABS*; Sheldrick, 2002)	*h* = −22→12
*T*_min_ = 0.847, *T*_max_ = 0.966	*k* = −6→6
13822 measured reflections	*l* = −27→28
4142 independent reflections	

### Refinement

**Table:**

Refinement on *F*^2^	Primary atom site location: structure-invariant direct methods
Least-squares matrix: full	Secondary atom site location: difference Fourier map
*R*[*F*^2^ > 2σ(*F*^2^)] = 0.049	Hydrogen site location: inferred from neighbouring sites
*wR*(*F*^2^) = 0.110	H-atom parameters constrained
*S* = 1.11	*w* = 1/[σ^2^(*F*_o_^2^) + (0.0377*P*)^2^ + 1.7007*P*] where *P* = (*F*_o_^2^ + 2*F*_c_^2^)/3
4142 reflections	(Δ/σ)_max_ < 0.001
273 parameters	Δρ_max_ = 0.34 e Å^−^^3^
0 restraints	Δρ_min_ = −0.40 e Å^−^^3^

### Special details

**Table:**

Geometry. All e.s.d.'s (except the e.s.d. in the dihedral angle between two l.s. planes) are estimated using the full covariance matrix. The cell e.s.d.'s are taken into account individually in the estimation of e.s.d.'s in distances, angles and torsion angles; correlations between e.s.d.'s in cell parameters are only used when they are defined by crystal symmetry. An approximate (isotropic) treatment of cell e.s.d.'s is used for estimating e.s.d.'s involving l.s. planes.
Refinement. Refinement of *F*^2^ against ALL reflections. The weighted *R*-factor *wR* and goodness of fit *S* are based on *F*^2^, conventional *R*-factors *R* are based on *F*, with *F* set to zero for negative *F*^2^. The threshold expression of *F*^2^ > σ(*F*^2^) is used only for calculating *R*-factors(gt) *etc*. and is not relevant to the choice of reflections for refinement. *R*-factors based on *F*^2^ are statistically about twice as large as those based on *F*, and *R*- factors based on ALL data will be even larger.

### Fractional atomic coordinates and isotropic or equivalent isotropic displacement parameters (Å^2^)

**Table:**

	*x*	*y*	*z*	*U*_iso_*/*U*_eq_	
Cl2	0.12456 (3)	−0.07231 (11)	−0.11690 (3)	0.01685 (14)	
Cl1	0.37576 (3)	0.54151 (11)	0.35382 (3)	0.01875 (14)	
O22	0.19785 (10)	−0.2141 (4)	−0.11310 (8)	0.0251 (4)	
O1B	0.07544 (11)	0.1702 (4)	0.02704 (8)	0.0275 (4)	
H1B	0.0786	0.0731	−0.0024	0.041*	
O42	0.09107 (12)	−0.1755 (4)	−0.06809 (9)	0.0330 (5)	
O21	0.42254 (13)	0.6540 (4)	0.31530 (9)	0.0369 (5)	
O31	0.35147 (11)	0.2777 (3)	0.33137 (9)	0.0300 (4)	
O41	0.30450 (11)	0.6993 (4)	0.34659 (8)	0.0264 (4)	
O2B	0.18407 (11)	−0.0563 (4)	0.08267 (8)	0.0282 (4)	
O32	0.14319 (11)	0.2034 (3)	−0.10376 (9)	0.0269 (4)	
O2A	0.31277 (11)	0.5865 (4)	0.15454 (8)	0.0287 (4)	
O11	0.42313 (11)	0.5324 (4)	0.42050 (8)	0.0269 (4)	
O1A	0.42090 (11)	0.3499 (4)	0.20638 (8)	0.0323 (5)	
H1A	0.4172	0.4352	0.2376	0.049*	
O12	0.06824 (11)	−0.1019 (4)	−0.17999 (8)	0.0274 (4)	
N2A	0.31083 (12)	0.1811 (4)	−0.01596 (9)	0.0189 (4)	
H2A	0.2759	0.2114	−0.0528	0.023*	
N2B	0.18027 (12)	0.3604 (4)	0.24939 (9)	0.0198 (4)	
H2B	0.2137	0.3306	0.2869	0.024*	
C3B	0.18684 (14)	0.2122 (4)	0.19911 (11)	0.0165 (5)	
N1B	0.24371 (13)	0.0283 (4)	0.21094 (9)	0.0225 (5)	
H11B	0.2751	0.0061	0.2496	0.027*	
H12B	0.2492	−0.0685	0.1799	0.027*	
C2B	0.13013 (14)	0.2659 (4)	0.13757 (11)	0.0156 (5)	
C2A	0.36459 (14)	0.2707 (5)	0.09571 (11)	0.0166 (5)	
N1A	0.24775 (12)	0.5022 (4)	0.02580 (9)	0.0204 (4)	
H11A	0.2145	0.5237	−0.0122	0.025*	
H12A	0.2432	0.5962	0.0576	0.025*	
C3A	0.30586 (14)	0.3245 (4)	0.03538 (11)	0.0160 (5)	
C5A	0.42317 (15)	−0.0621 (5)	0.04338 (12)	0.0239 (5)	
H5A	0.4618	−0.1915	0.0457	0.029*	
C6A	0.42192 (15)	0.0799 (5)	0.09851 (12)	0.0213 (5)	
H6A	0.4605	0.0447	0.1378	0.026*	
C4A	0.36681 (15)	−0.0065 (5)	−0.01335 (12)	0.0223 (5)	
H4A	0.3666	−0.0978	−0.0507	0.027*	
C6B	0.07394 (14)	0.4623 (5)	0.13287 (11)	0.0200 (5)	
H6B	0.0373	0.4991	0.0927	0.024*	
C1A	0.36249 (15)	0.4192 (5)	0.15434 (11)	0.0199 (5)	
C1B	0.13334 (15)	0.1106 (5)	0.08066 (11)	0.0183 (5)	
C4B	0.12476 (15)	0.5518 (5)	0.24461 (12)	0.0229 (5)	
H4B	0.1238	0.6456	0.2813	0.027*	
C5B	0.07058 (15)	0.6081 (5)	0.18698 (12)	0.0222 (5)	
H5B	0.0322	0.7396	0.1833	0.027*	

### Atomic displacement parameters (Å^2^)

**Table:**

	*U*^11^	*U*^22^	*U*^33^	*U*^12^	*U*^13^	*U*^23^
Cl2	0.0180 (3)	0.0159 (3)	0.0166 (3)	0.0000 (2)	0.0052 (2)	−0.0011 (2)
Cl1	0.0213 (3)	0.0159 (3)	0.0199 (3)	−0.0054 (2)	0.0074 (2)	−0.0038 (2)
O22	0.0216 (9)	0.0260 (10)	0.0251 (9)	0.0067 (7)	0.0028 (7)	−0.0018 (7)
O1B	0.0229 (10)	0.0384 (11)	0.0194 (9)	0.0070 (8)	0.0036 (7)	−0.0085 (8)
O42	0.0463 (12)	0.0321 (11)	0.0289 (10)	−0.0153 (9)	0.0242 (9)	−0.0079 (8)
O21	0.0472 (13)	0.0380 (12)	0.0356 (11)	−0.0184 (10)	0.0275 (10)	−0.0084 (9)
O31	0.0327 (11)	0.0164 (9)	0.0365 (11)	−0.0060 (8)	0.0034 (9)	−0.0079 (8)
O41	0.0268 (10)	0.0233 (9)	0.0271 (9)	0.0037 (8)	0.0050 (8)	−0.0032 (8)
O2B	0.0348 (11)	0.0259 (10)	0.0228 (9)	0.0121 (8)	0.0067 (8)	−0.0040 (8)
O32	0.0322 (11)	0.0143 (9)	0.0295 (10)	0.0002 (7)	0.0019 (8)	−0.0008 (7)
O2A	0.0334 (11)	0.0286 (10)	0.0225 (9)	0.0122 (8)	0.0058 (8)	−0.0031 (8)
O11	0.0237 (10)	0.0305 (10)	0.0227 (9)	−0.0036 (8)	0.0013 (7)	−0.0032 (8)
O1A	0.0249 (10)	0.0511 (13)	0.0176 (9)	0.0117 (9)	0.0010 (8)	−0.0044 (9)
O12	0.0232 (9)	0.0331 (11)	0.0214 (9)	0.0054 (8)	−0.0005 (7)	−0.0064 (8)
N2A	0.0215 (11)	0.0188 (10)	0.0156 (9)	0.0020 (8)	0.0044 (8)	0.0008 (8)
N2B	0.0230 (11)	0.0192 (10)	0.0165 (9)	−0.0029 (8)	0.0047 (8)	−0.0026 (8)
C3B	0.0185 (12)	0.0137 (11)	0.0182 (11)	−0.0045 (9)	0.0071 (9)	−0.0022 (9)
N1B	0.0279 (12)	0.0187 (10)	0.0182 (10)	0.0048 (9)	0.0024 (8)	−0.0005 (8)
C2B	0.0167 (12)	0.0127 (10)	0.0186 (11)	−0.0040 (9)	0.0071 (9)	−0.0013 (9)
C2A	0.0149 (12)	0.0162 (11)	0.0190 (11)	−0.0007 (9)	0.0055 (9)	0.0020 (9)
N1A	0.0227 (11)	0.0177 (10)	0.0178 (9)	0.0073 (8)	0.0012 (8)	−0.0002 (8)
C3A	0.0177 (12)	0.0125 (10)	0.0186 (11)	−0.0025 (9)	0.0067 (9)	0.0012 (9)
C5A	0.0213 (13)	0.0204 (12)	0.0326 (13)	0.0071 (10)	0.0120 (11)	0.0006 (11)
C6A	0.0187 (12)	0.0225 (12)	0.0222 (12)	0.0015 (10)	0.0050 (10)	0.0044 (10)
C4A	0.0267 (13)	0.0174 (12)	0.0260 (12)	0.0003 (10)	0.0127 (10)	−0.0035 (10)
C6B	0.0179 (12)	0.0187 (12)	0.0230 (11)	−0.0025 (10)	0.0053 (9)	−0.0005 (10)
C1A	0.0219 (13)	0.0201 (12)	0.0176 (11)	−0.0015 (10)	0.0056 (10)	0.0014 (9)
C1B	0.0201 (12)	0.0169 (12)	0.0183 (11)	−0.0018 (10)	0.0062 (9)	−0.0009 (9)
C4B	0.0279 (14)	0.0193 (12)	0.0251 (12)	−0.0043 (11)	0.0135 (10)	−0.0075 (10)
C5B	0.0213 (13)	0.0174 (12)	0.0302 (13)	0.0012 (10)	0.0112 (11)	−0.0047 (10)

### Geometric parameters (Å, º)

**Table:**

Cl2—O12	1.4316 (17)	C3B—C2B	1.428 (3)
Cl2—O22	1.4427 (18)	N1B—H11B	0.86
Cl2—O42	1.4463 (18)	N1B—H12B	0.86
Cl2—O32	1.4466 (18)	C2B—C6B	1.378 (3)
Cl1—O11	1.4336 (17)	C2B—C1B	1.477 (3)
Cl1—O21	1.4427 (19)	C2A—C6A	1.378 (3)
Cl1—O41	1.4430 (18)	C2A—C3A	1.424 (3)
Cl1—O31	1.4451 (18)	C2A—C1A	1.485 (3)
O1B—C1B	1.325 (3)	N1A—C3A	1.324 (3)
O1B—H1B	0.82	N1A—H11A	0.86
O2B—C1B	1.214 (3)	N1A—H12A	0.86
O2A—C1A	1.212 (3)	C5A—C4A	1.353 (3)
O1A—C1A	1.320 (3)	C5A—C6A	1.399 (3)
O1A—H1A	0.82	C5A—H5A	0.93
N2A—C4A	1.350 (3)	C6A—H6A	0.93
N2A—C3A	1.352 (3)	C4A—H4A	0.93
N2A—H2A	0.86	C6B—C5B	1.401 (3)
N2B—C4B	1.351 (3)	C6B—H6B	0.93
N2B—C3B	1.355 (3)	C4B—C5B	1.351 (3)
N2B—H2B	0.86	C4B—H4B	0.93
C3B—N1B	1.328 (3)	C5B—H5B	0.93
			
O12—Cl2—O22	110.08 (10)	C3A—C2A—C1A	119.5 (2)
O12—Cl2—O42	110.43 (12)	C3A—N1A—H11A	120
O22—Cl2—O42	108.41 (12)	C3A—N1A—H12A	120
O12—Cl2—O32	109.81 (11)	H11A—N1A—H12A	120
O22—Cl2—O32	109.27 (11)	N1A—C3A—N2A	118.0 (2)
O42—Cl2—O32	108.81 (11)	N1A—C3A—C2A	125.4 (2)
O11—Cl1—O21	109.92 (11)	N2A—C3A—C2A	116.5 (2)
O11—Cl1—O41	110.16 (11)	C4A—C5A—C6A	118.4 (2)
O21—Cl1—O41	109.17 (12)	C4A—C5A—H5A	120.8
O11—Cl1—O31	109.34 (11)	C6A—C5A—H5A	120.8
O21—Cl1—O31	109.36 (12)	C2A—C6A—C5A	121.2 (2)
O41—Cl1—O31	108.87 (11)	C2A—C6A—H6A	119.4
C1B—O1B—H1B	109.5	C5A—C6A—H6A	119.4
C1A—O1A—H1A	109.5	N2A—C4A—C5A	120.2 (2)
C4A—N2A—C3A	124.5 (2)	N2A—C4A—H4A	119.9
C4A—N2A—H2A	117.8	C5A—C4A—H4A	119.9
C3A—N2A—H2A	117.8	C2B—C6B—C5B	121.7 (2)
C4B—N2B—C3B	124.5 (2)	C2B—C6B—H6B	119.2
C4B—N2B—H2B	117.8	C5B—C6B—H6B	119.2
C3B—N2B—H2B	117.8	O2A—C1A—O1A	123.5 (2)
N1B—C3B—N2B	118.1 (2)	O2A—C1A—C2A	123.8 (2)
N1B—C3B—C2B	125.6 (2)	O1A—C1A—C2A	112.7 (2)
N2B—C3B—C2B	116.3 (2)	O2B—C1B—O1B	123.0 (2)
C3B—N1B—H11B	120	O2B—C1B—C2B	123.3 (2)
C3B—N1B—H12B	120	O1B—C1B—C2B	113.6 (2)
H11B—N1B—H12B	120	C5B—C4B—N2B	120.6 (2)
C6B—C2B—C3B	119.0 (2)	C5B—C4B—H4B	119.7
C6B—C2B—C1B	121.7 (2)	N2B—C4B—H4B	119.7
C3B—C2B—C1B	119.3 (2)	C4B—C5B—C6B	118.0 (2)
C6A—C2A—C3A	119.1 (2)	C4B—C5B—H5B	121
C6A—C2A—C1A	121.3 (2)	C6B—C5B—H5B	121
			
C4B—N2B—C3B—N1B	−179.2 (2)	C3A—N2A—C4A—C5A	−0.1 (4)
C4B—N2B—C3B—C2B	−0.3 (3)	C6A—C5A—C4A—N2A	−0.3 (4)
N1B—C3B—C2B—C6B	179.4 (2)	C3B—C2B—C6B—C5B	−0.7 (4)
N2B—C3B—C2B—C6B	0.7 (3)	C1B—C2B—C6B—C5B	179.5 (2)
N1B—C3B—C2B—C1B	−0.8 (4)	C6A—C2A—C1A—O2A	−179.1 (2)
N2B—C3B—C2B—C1B	−179.6 (2)	C3A—C2A—C1A—O2A	0.4 (4)
C4A—N2A—C3A—N1A	−180.0 (2)	C6A—C2A—C1A—O1A	1.1 (3)
C4A—N2A—C3A—C2A	0.2 (3)	C3A—C2A—C1A—O1A	−179.4 (2)
C6A—C2A—C3A—N1A	−179.8 (2)	C6B—C2B—C1B—O2B	176.9 (2)
C1A—C2A—C3A—N1A	0.7 (4)	C3B—C2B—C1B—O2B	−2.8 (4)
C6A—C2A—C3A—N2A	0.0 (3)	C6B—C2B—C1B—O1B	−3.2 (3)
C1A—C2A—C3A—N2A	−179.6 (2)	C3B—C2B—C1B—O1B	177.1 (2)
C3A—C2A—C6A—C5A	−0.3 (4)	C3B—N2B—C4B—C5B	0.0 (4)
C1A—C2A—C6A—C5A	179.2 (2)	N2B—C4B—C5B—C6B	0.0 (4)
C4A—C5A—C6A—C2A	0.5 (4)	C2B—C6B—C5B—C4B	0.4 (4)

### Hydrogen-bond geometry (Å, º)

**Table:**

*D*—H···*A*	*D*—H	H···*A*	*D*···*A*	*D*—H···*A*
O1*A*—H1*A*···O21	0.82	1.99	2.810 (3)	173
O1*B*—H1*B*···O42	0.82	1.96	2.779 (3)	176
N2*A*—H2*A*···O32	0.86	2.24	2.968 (3)	142
N2*A*—H2*A*···O41^i^	0.86	2.41	3.004 (3)	126
N2*B*—H2*B*···O31	0.86	2.31	3.005 (3)	138
N2*B*—H2*B*···O41	0.86	2.54	3.057 (3)	120
N2*B*—H2*B*···O22^ii^	0.86	2.34	2.992 (3)	133
N1*A*—H11*A*···O22^iii^	0.86	2.50	3.211 (3)	141
N1*A*—H11*A*···O32	0.86	2.58	3.231 (3)	133
N1*B*—H11*B*···O31	0.86	2.32	3.000 (3)	136
N1*B*—H11*B*···O41^iv^	0.86	2.54	3.268 (3)	143
N1*A*—H12*A*···O2*A*	0.86	2.09	2.711 (3)	129
N1*A*—H12*A*···O2*B*^iii^	0.86	2.19	2.928 (3)	144
N1*B*—H12*B*···O2*A*^iv^	0.86	2.22	2.971 (3)	145
N1*B*—H12*B*···O2*B*	0.86	2.07	2.693 (3)	128
C4*A*—H4*A*···O11^v^	0.93	2.57	3.312 (3)	137
C4*B*—H4*B*···O32^vi^	0.93	2.53	3.433 (3)	165
C5*A*—H5*A*···O11^vii^	0.93	2.37	3.277 (3)	164
C5*B*—H5*B*···O12^viii^	0.93	2.52	3.450 (3)	177
C6*A*—H6*A*···O1*A*	0.93	2.38	2.711 (3)	100
C6*B*—H6*B*···O1*B*	0.93	2.41	2.735 (3)	100

Symmetry codes: (i) *x*, −*y*+1, *z*−1/2; (ii) *x*, −*y*, *z*+1/2; (iii) *x*, *y*+1, *z*; (iv) *x*, *y*−1, *z*; (v) *x*, −*y*, *z*−1/2; (vi) *x*, −*y*+1, *z*+1/2; (vii) −*x*+1, *y*−1, −*z*+1/2; (viii) −*x*, −*y*+1, −*z*.
